# GLP-1 receptor agonist use and overall survival among women with type 2 diabetes and breast cancer: a retrospective cohort study

**DOI:** 10.1093/oncolo/oyag268

**Published:** 2026-07-11

**Authors:** Rebecca A Nelson, Ying-Chuen Lai, Sung H Kil, Hayley S Lee, Joanne Mortimer, Ping H Wang

**Affiliations:** Department of Computational and Quantitative Medicine, Beckman Research Institute, City of Hope, Duarte, CA, 91010, United States; Department of Internal Medicine, National Taiwan University Hospital, Taipei City, 100229, Taiwan; Department of Diabetes, Endocrinology, and Metabolism, City of Hope National Medical Center, Duarte, CA, 91010, United States; Arthur Riggs Diabetes & Metabolism Research Institute, Beckman Research Institute, City of Hope, Duarte, CA, 91010, United States; Arthur Riggs Diabetes & Metabolism Research Institute, Beckman Research Institute, City of Hope, Duarte, CA, 91010, United States; Department of Medical Oncology & Therapeutics Research, City of Hope National Medical Center, Duarte, CA, 91010, United States; Department of Diabetes, Endocrinology, and Metabolism, City of Hope National Medical Center, Duarte, CA, 91010, United States

**Keywords:** glucagon-like peptide-1 receptor agonists, diabetes mellitus, breast neoplasms, mortality

## Abstract

**Background:**

Diabetes and obesity are associated with worse prognosis in women with breast cancer. However, the impact of glucagon-like peptide-1 receptor agonists (GLP-1RA) on survival following breast cancer diagnosis remains unclear.

**Methods:**

We conducted a single institution, propensity-score matched, retrospective, 2-year landmark study of women with type 2 diabetes (T2D) diagnosed with breast cancer at City of Hope (2009-2025). Patients were categorized based on post-diagnosis exposure to GLP-1RA. Patients were compared across exposure groups using t-tests for continuous variables, chi-square tests for categorical variables, multivariable Cox proportional hazards models for overall mortality risk, and Kaplan-Meier plots for survival time.

**Results:**

In 226 matched pairs, there were 47 deaths (30 in unexposed patients and 17 in exposed patients). GLP-1RA exposure was associated with a significantly lower risk of overall mortality (HR = 0.51, 95% CI 0.28-0.93, *P* = .03) on multivariable analysis. A sensitivity analysis in stage I-III patients yielded similar results (HR = 0.45, 95% CI 0.23-0.85, *P* = .01). Kaplan-Meier survival analysis also showed an association between increased overall survival and GLP-1RA exposure (log-rank *P* = .02).

**Conclusions:**

In this retrospective cohort of women with T2D and breast cancer, GLP-1RA use after diagnosis was associated with improved overall survival in T2D. These findings reflect all-cause mortality and do not distinguish between cancer-specific and non-cancer causes of death and should therefore be interpreted accordingly.

## Introduction

Type 2 diabetes (T2D) and obesity are associated with increased breast cancer risk and poorer outcomes.^1^^,^^2^ Patients with T2D have been shown to experience shorter survival following a breast cancer diagnosis, potentially reflecting both cancer-related and competing cardiometabolic risks.^3^ Glucagon-like peptide-1 receptor agonists (GLP-1RA) are widely used to treat T2D and obesity and have demonstrated substantial cardiometabolic benefits.^4^ However, their impact on survival following a breast cancer diagnosis remains unclear. Data evaluating GLP-1RA exposure in patients with coexisting breast cancer and T2D, particularly in real-world settings, are limited.^5^ In this retrospective study, we evaluated the association between post-diagnosis GLP-1RA use and overall survival among women with T2D and breast cancer treated at City of Hope (COH).

## Methods

### Patient selection

Women with T2D diagnosed with invasive breast cancer between 2009-2025 were identified using the COH Cancer Registry, which align with the approval of GLP-1RA for T2D (first approval in 2010).

Patients were eligible if medication information was documented in the medical record, and complete data were available on all covariates of interest (Figure S1). Only medications captured within the medical records were identified. Among 1281 eligible patients, 461 (36%) had documented GLP-1RA exposure after their breast cancer diagnosis while 820 (64%) did not.

### Landmark criteria and patient matching

To mitigate potential immortal time bias, we conducted a landmark analysis using a prespecified landmark of 2 years after breast cancer diagnosis, corresponding to the median time from diagnosis to first GLP-1RA initiation among exposed patients. Patients who died or had insufficient follow-up prior to the landmark were excluded. Propensity score matching (1:1) was used to balance demographic and clinical characteristics between exposure groups, including age, diagnosis year, race, ethnicity, BMI, tumor stage and grade, ER/PR and HER2 status, and treatment modalities (surgery, endocrine therapy, radiotherapy, and chemotherapy). The propensity score model used nearest neighbor matching without replacement, a caliper width of 0.25, and a generalized linear model for the distance metric. A Love plot was generated to illustrate covariable balance (Figure S2). To further minimize immortal time bias, matched pairs were excluded if the unexposed patient had shorter follow-up than the exposed patient’s time to GLP-1RA exposure.

### Statistical analysis

Baseline characteristics were compared using t-tests and chi-square tests. A bidirectional stepwise Cox regression (entry and retention criteria of 0.15) was used to identify relevant covariates. The final Cox model included GLP-1RA exposure, diagnosis age, diagnosis year, and cancer stage. A sensitivity analysis was performed excluding Stage IV patients to reduce potential confounding by advanced disease burden and differential survival trajectories. Survival was estimated using Kaplan-Meier methods and compared using the log-rank test. Follow-up time was calculated from the date of diagnosis to the date of last contact, with patients censored at their last known follow-up.

This study was approved by the COH Institutional Review Board. All analyses were conducted using SAS (version 9.4, SAS Institute Inc) or R (version 4.2.0, https://www.r-project.org/). Significance was set at a 2-sided alpha of 0.05.

## Results

**Figure 1.**
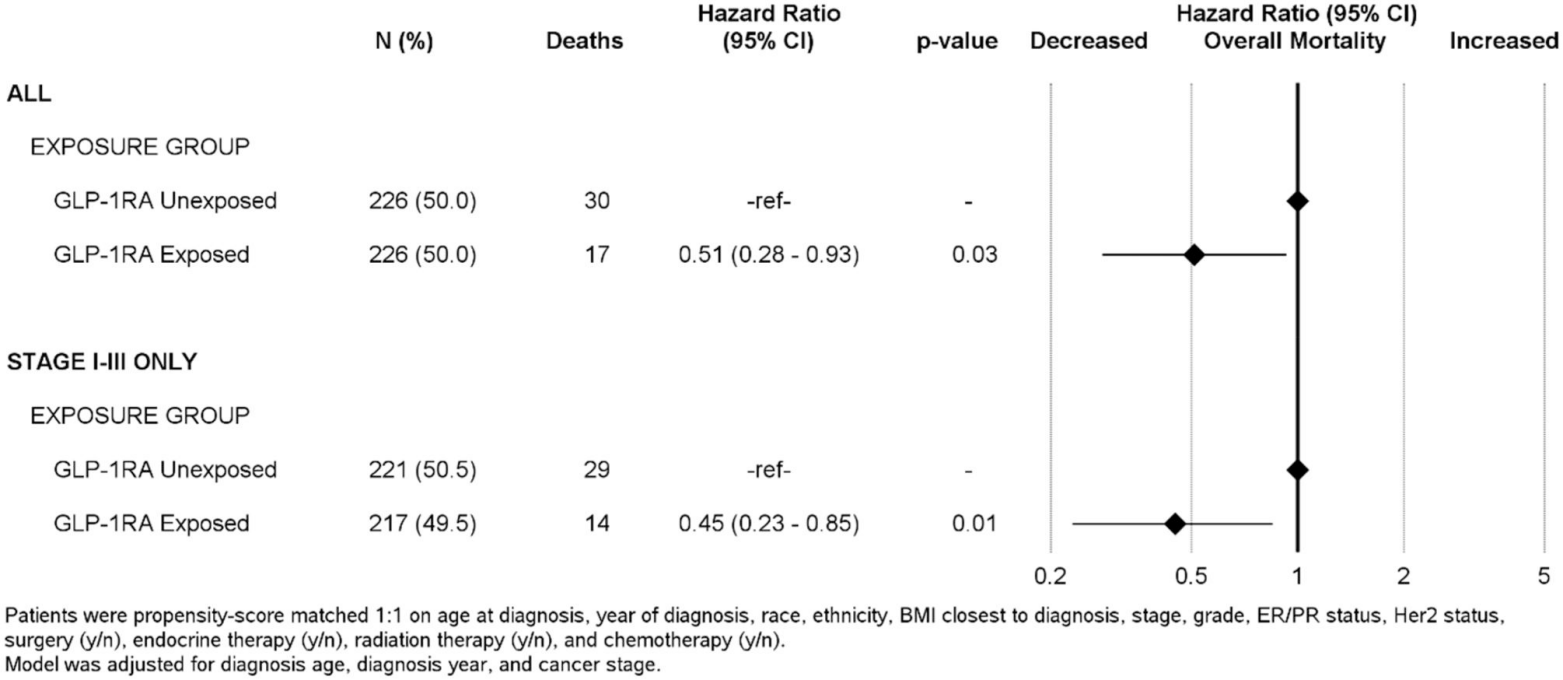
HR estimates and 95% CIs for a multivariable Cox proportional hazard model of factors associated with overall mortality in a 1:1 propensity-score matched cohort of type II diabetic women with breast cancer at City of Hope, 2009-2025. Two models were run: (i) all patients, and (ii) stage I to III only patients. Both multivariable models were adjusted for diagnosis age, diagnosis year, and cancer stage. Abbreviations: HR: hazard ratio; 95% CI: confidence interval.

**Figure 2.**
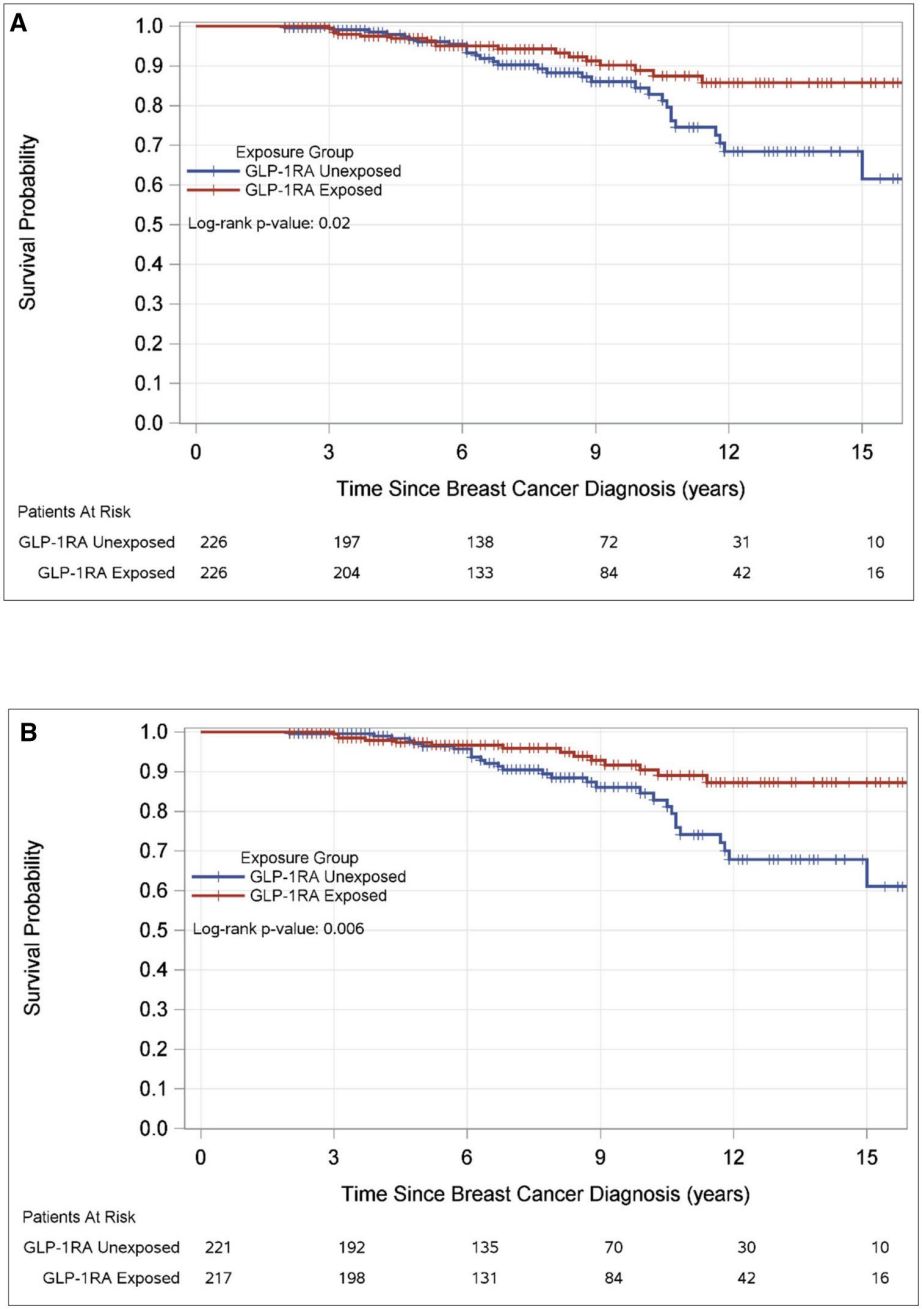
(A) Kaplan-Meier survival plots depicting overall survival by GLP-1RA exposure status measured from cancer diagnosis in a 1:1 propensity-score matched cohort of type II diabetic women with breast cancer at City of Hope, 2009-2025. (B) Kaplan-Meier survival plots depicting overall survival by GLP-1RA exposure status measured from cancer diagnosis in a 1:1 propensity-score matched cohort of type II diabetic women with stage I to III breast cancer at City of Hope, 2009-2025.

A total of 452 women with T2D and invasive breast cancer diagnosed between 2009-2025 were included (*n* = 226 matched pairs). Baseline characteristics were well balanced after matching, with minor differences in diagnosis year and BMI that were not clinically meaningful (Table S1). In multivariable Cox models, GLP-1RA exposure was associated with decreased overall mortality (HR = 0.51, 95% CI 0.26-0.93, *P* = .03) (Figure 1). A sensitivity analysis in stage I-III patients yielded similar results (HR = 0.45, 95% CI 0.23-0.85, *P* = .01). Kaplan-Meier results also showed an association between increased overall survival and GLP-1RA exposure in all patients (log rank *P* = .02) as well as stage I-III patients (log-rank = 0.006) (Figure 2).

## Discussion

In this single-center retrospective study, post-diagnosis use of GLP-1RA in women with T2D and breast cancer diagnosis was associated with improved overall survival. Importantly, this endpoint reflects all-cause survival and does not allow for distinction between breast cancer specific and non-cancer causes of death.

Clinical trials have demonstrated that GLP-1RA therapy reduces adverse cardiovascular events and cardiovascular mortality in patients with T2D and obesity, suggesting that improved cardiometabolic health may play an important role in the observed survival benefit.^6^^,^^7^ The observed overall mortality in our study likely captures both cancer-related and competing causes of mortality. Our findings are consistent with a large cohort study of 841 831 women with breast cancer, which reported improved overall survival and reduced cancer recurrence among GLP-1RA users with T2D compared with users of other antidiabetic medications.^8^ However, cardiovascular outcomes were not systematically captured in our cancer registry, precluding direct evaluation of these effects.

Prior studies have shown that higher GLP-1 receptor gene expression in cancer tissues is associated with improved overall survival, suggesting a possible tumor-level mechanism.^9^ Other studies highlighted biologic pathways linking diabetes and obesity to tumor progression, including chronic inflammation, dysregulated adipocytokines and insulin-like growth factor-1 signaling, which may be modified by GLP-1RA therapy^10^ These mechanisms remain speculative in the context of this study and require further investigation.

### Strengths and limitations

Strengths of our study include a well-characterized cohort within a specialized cancer center, standardized abstraction of tumor-specific data by trained cancer registrars, and long-term follow-up for vital status. Several limitations should be considered. First, as a retrospective observational study, residual confounding cannot be excluded, and causal inference is limited. Second, although vital status follow-up is current, it often captures death without reliable attribution to specific causes. Third, recurrence data were not consistently captured, particularly for events occurring outside our institution, precluding analysis of recurrence-free or breast cancer-specific outcomes. Fourth, information on medication adherence was unavailable. Collectively, these limitations restrict interpretation to associations with overall survival and do not allow differentiation between cancer-related and competing risks.

## Conclusion

In this retrospective cohort of women with T2D and breast cancer, post-diagnosis GLP-1RA use was associated with improved overall survival. Prospective studies with comprehensive capture of cause-specific mortality, recurrence, and cardiometabolic outcomes are needed to clarify the underlying mechanisms and determine whether GLP-1RA use is associated with breast cancer-specific benefit.

## Supplementary Material

oyag268_Supplementary_Data

## Data Availability

The de-identified raw data will be available with publication and can be obtained from the corresponding author upon reasonable request and subject to institutional review board approval and a data use agreement.
