# Supplementary material for: GLP-1 receptor agonist use and overall survival among women with type 2 diabetes and breast cancer: a retrospective cohort study
Source: Oncologist. 2026 Jul 11;31(8):oyag268. doi: 10.1093/oncolo/oyag268 (PMC13395082; doi:10.1093/oncolo/oyag268)
Supplement: oyag268_Supplementary_Data [file oyag268_supplementary_data.docx]

**Table S1**. Comparison of baseline characteristics by GLP-1RA exposure status in a 1:1 matched* cohort of T2D women with breast cancer at City of Hope, 2009-2025.

|  | | **GLP-1RA Unexposed (Total N=226)** | **GLP-1RA Exposed (Total N=226)** | **p-value** |  |
| --- | --- | --- | --- | --- | --- |
| Age | Median (IQR^+^) | 58 (51 - 65) | 58 (49 - 64) | 0.36 |  |
| Age Group | <55 | 87 (38.5) | 89 (39.4) | 0.85 |  |
|  | 55+ | 139 (61.5) | 137 (60.6) |  |  |
| Diagnosis Year | Median (IQR^+^) | 2018 (2015 - 2021) | 2018 (2014 - 2020) | 0.61 |  |
| Race | White | 166 (73.5) | 168 (74.3) | 0.84 |  |
|  | Black | 23 (10.2) | 22 (9.7) |  |  |
|  | API | 37 (16.4) | 36 (15.9) |  |  |
| Ethnicity | Non-Hispanic | 125 (55.3) | 128 (56.6) | 0.78 |  |
|  | Hispanic | 101 (44.7) | 98 (43.4) |  |  |
| Closest BMI to Dx | Median (IQR^+^) | 31.4 (27.6 - 35) | 31.9 (28.3 - 36.7) | 0.10 |  |
| Tumor Grade | I | 21 (9.3) | 20 (8.8) | 0.76 |  |
|  | II | 116 (51.3) | 114 (50.4) |  |  |
|  | III | 89 (39.4) | 92 (40.7) |  |  |
| Cancer Stage | I | 102 (45.1) | 99 (43.8) | 0.61 |  |
|  | II | 84 (37.2) | 82 (36.3) |  |  |
|  | III | 35 (15.5) | 36 (15.9) |  |  |
|  | IV | 5 (2.2) | 9 (4.0) |  |  |
| ER/PR Status | Positive | 191 (84.5) | 185 (81.9) | 0.45 |  |
|  | Negative | 35 (15.5) | 41 (18.1) |  |  |
| Her2 Status | Positive | 50 (22.1) | 45 (19.9) | 0.56 |  |
|  | Negative | 176 (77.9) | 181 (80.1) |  |  |
| Received Surgery | Yes | 216 (95.6) | 214 (94.7) | 0.66 |  |
|  | No | 10 (4.4) | 12 (5.3) |  |  |
| Endocrine Therapy | Yes | 165 (73.0) | 163 (72.1) | 0.83 |  |
|  | No | 61 (27.0) | 63 (27.9) |  |  |
| Radiotherapy | Yes | 130 (57.5) | 141 (62.4) | 0.29 |  |
|  | No | 96 (42.5) | 85 (37.6) |  |  |
| Chemotherapy | Yes | 133 (58.8) | 143 (63.3) | 0.34 |  |
|  | No | 93 (41.2) | 83 (36.7) |  |  |
| Abbreviations: BMI: body mass index, kg/cm^2; ER: estrogen receptor; HER2: human epidermal growth factor receptor 2; IQR: interquartile range; PR: progesterone receptor; SD: standard deviation. *Patients were matched 1:1 on age at diagnosis, year of diagnosis, race, ethnicity, BMI closest to diagnosis, stage, grade, ER/PR status, Her2 status, surgery (y/n), endocrine therapy (y/n), radiation therapy (y/n), and chemotherapy (y/n). Median follow-up since breast cancer diagnosis was 6.8 (IQR: 4.2 - 9.9) years for GLP-1RA unexposed patients and 6.9 (IQR: 4.5 - 10.8) years for GLP-1RA exposed patients. There were 30 (13.3%) deaths in GLP-1RA unexposed patients and 17 (7.5%) in GLP-1RA exposed patients. | | | | | |
